# Cognitive cerebellum dominates motor cerebellum in functional decline of older adults with mild cognitive impairment

**DOI:** 10.1371/journal.pone.0321304

**Published:** 2025-04-03

**Authors:** Maria Devita, Alessandra Coin, Chiara Ceolin, Giulia Debiasi, Mariagiulia Anglani, Chiara Begliomini, Simone Cauzzo, Francesca Biasin, Carlo Gabelli, Alessandra Codemo, Michela Sarlo, Marina De Rui, Giuseppe Sergi, Alessandra Bertoldo, Daniela Mapelli

**Affiliations:** 1 Department of General Psychology (DPG), University of Padua, Padua, Italy; 2 Geriatrics Unit, Department of Medicine (DIMED), University of Padua, Padua, Italy; 3 Department of Neurobiology, Care Sciences and Society, Karolinska Institutet and Stockholm University, Aging Research Center, Stockholm, Sweden; 4 Department of Surgery, Oncology and Gastroenterology, University of Padua, Padua, Italy; 5 Department of Information Engineering, University of Padua, Padua, Italy; 6 Neuroradiology Unit, University Hospital of Padua, Padua, Italy; 7 Padova Neuroscience Center, University of Padua, Padua, Italy; 8 Parkinson’s Disease and Movement Disorders Unit, Center for Rare Neurological Diseases, Department of Neurosciences, University of Padova, Padua, Italy; 9 Geriatrics Unit, Ospedale San Bortolo, Vicenza, Italy; 10 Regional Brain Aging Centre, University of Padua, Padua, Italy; 11 Department of Communication Sciences, Humanities and International Studies, University of Urbino Carlo Bo, Urbino, Italy; Isfahan University of Medical Sciences, IRAN, ISLAMIC REPUBLIC OF

## Abstract

**Objectives:**

The present study aims to investigate the role that cognitive cerebellar lobules, compared to the motor ones, could have on performance abilities control in older individuals with Mild Cognitive Impairment (MCI).

**Methods:**

Thirty-six participants with MCI were retrospectively recruited from the outpatient clinic for Cognitive Decline and Dementia at Geriatric Clinic and Regional Center for Brain Aging.

Cognition was assessed through a reaction time (RT) task in which a mere cognitive (COG) component (RT/S1 COG, RT/S3 COG) has been isolated from a motor (MOT) component (RT/S1 MOT, RT/S3 MOT). Performance abilities were evaluated using Short Physical Performance Battery (SPPB), Tinetti Scale, and Activities of Daily Living (ADL). Finally, structural neuroimaging was conducted using magnetic resonance imaging at 3T.

**Results:**

Left_Crus_I showed a correlation with SPPB, ADL%, and RT/S3 COG. Vermis_VI and Right_VI were correlated to ADL%, and RT/S3 COG with the entire lobule VI. ADL% showed negative correlations with RT/S1 COG, RT/S3 COG, and RT/S3 MOT. In the regression analysis, the strongest associations were found between RT/S3 COG and SPPB gait speed (R^2^ = 0.44, p = 0.03), Tinetti gait speed (R^2^ = 0.62, p < 0.001), and ADL% (R^2^ = 0.78, p < 0.001). Regarding cerebellar volumes, Right_Crus_I was associated with all SPPB tests, while Left_VI was associated with functional autonomy (ADL%: R^2^ = 0.78, p = 0.04). No associations were found between performance variables and total intracranial volume.

**Conclusions:**

This study highlights that the cognitive cerebellar component dominates over the motor one even in the control of physical and functional capabilities of older adults with MCI.

## 1. Introduction

Postural control is defined as the ability to maintain or regain balance during both static and dynamic activities [1,2]. This capability is crucial for older adults as it is intricately involved in numerous daily tasks such as walking, dressing, or driving [1,2]. The complexity of movement and coordination tasks relies on efficient cognitive control, given postural regulation involves the integration of somatosensory, neuromuscular, and proprioceptive systems [3]. Reaction times (RTs), which are often used to evaluate cognitive processing, can be a valuable assessment tool also in motor tasks, as they may be indicative of the underlying cognitive processes involved in movement planning and execution. Welford distinguished three components of RTs: the first is “the time taken by the stimulus to activate the sense organ and for impulses to travel from it to the brain”; the second component is related to the central processes that allow to detect the target and to start a response. Finally, the third component represents the “time required to energize the muscles and to produce an overt recordable response” [4]. Some other studies supported the hypothesis of cognitive (central) and motor (peripheral) as independent components of RTs, by measuring separately the time required to initiate a response (decision time) and the time required to give the appropriate motor reaction to a stimulus [5–7]. In their study, Danev et al. also suggested that RTs are strongly influenced by vigilance, with longer cognitive and motor reaction times should be expected in association with low levels of vigilance [5].

In this study, the implementation of a specific computerized methodology allowed us to distinguish between cognitive and motor components of RTs, enhancing our understanding of various levels of information processing and its association with cerebellar lobules and physical/performance abilities. More in detail, the cognitive component of RTs is represented by the time occurring between the appearance of the target and the moment the participant’s finger leaves the rest button. The motor component is, instead, conceptualized as the time occurring between the finger leaving the rest button and the reaction button being pressed in response to the target. In other words, the target stimulus must be processed through visuospatial attention mechanisms (including stimulus encoding and decision-making processes - cognitive RTs); secondly, an appropriate motor response needs to be selected and executed (motor RTs).

The decline in both motor/cognitive functioning and in processing speed, as individuals age raises the risk of falls, fractures, and disabilities, consequently amplifying overall frailty among those with clinical cognitive impairment [8]. This may partly stems from shared mechanisms in both these domains, including neurodegenerative damage, chronic inflammation, metabolic/oxidative stress, as well as lifestyle and psychosocial factors [3,9,10]. These processes not only affect the brain but also the cerebellum. Traditionally recognized as a key structure for motor coordination, the cerebellum has recently been acknowledged to play a role in cognitive functions [11]. This view suggests that, in the case of a neurodegenerative disease affecting the central nervous system, the cerebellum could also be involved to a similar extent as the brain. Consequently, it could significantly contribute to shaping the cognitive deficit profile that is usually observed in these cases. Previous studies have shown that larger grey matter volumes are correlated with faster gait speed [12]. However, the literature on this topic remains limited, particularly regarding the specific roles of cerebellar lobules and their potential contributions to the cognitive modulation of movement and balance, as well as functional independence in aging populations.

The hypothesis of the present study is that physical and performance tests, well-known to be specifically mediated by motor control, could be driven by cognitive functioning too, revealing a novel and unexpected relationship with cerebellar lobules implicated in cognitive functioning.

Therefore, our aim is to investigate the relationship between physical and performance abilities, cerebellar volume and reaction times in older individuals with mild cognitive impairment (MCI). The methodology employed to differentiate the various components of RTs is crucial and innovative for elucidating the roles of the cognitive cerebellum versus the motor cerebellum in the functional decline observed in this population. As mentioned before, although the cerebellum has traditionally been linked primarily to motor functions, there remains limited evidence regarding its role in cognitive modulation. Analyzing the cognitive and motor components of RTs separately may be pivotal for enhancing our understanding of the cerebellum’s enigmatic cognitive functions, even in contexts where such involvement is not expected (e.g., predominantly motor tasks).

## 2. Materials and methods

This is a retrospective observational study; participants were recruited among those accessing the outpatient clinic for Cognitive Decline and Dementia at the Geriatric Clinic and the Regional Center for Brain Aging, University Hospital of Padua. All individuals gave their informed consent and were involved on a voluntary basis. The inclusion and exclusion criteria of participants, as well as the clinical and neurological assessments, were detailed in a prior publication [13]. The data were accessed for research purposes on June 15, 2023. The authors did not have access to information that could identify the participants during or after data collection. Additional assessments employed in these analyses include:

*Reaction times test:* The RT/S1 is a computerized test designed to assess the speed of information processing, covering both cognitive and motor reaction times [14]. Participants are required to press a button when a yellow light appears on the screen. It starts with five practice stimuli, followed by 28 test stimuli, taking about seven minutes. The test measures Cognitive Reaction Time (RT/S1 COG) and Motor Reaction Time (RT/S1 MOT). RT/S1 COG is the time (in ms) from the target’s appearance to lifting the finger from the rest button, while RT/S1 MOT is the time from lifting the finger to pressing the reaction button. The RT/S3 test is a more advanced version, featuring yellow and red lights with a tone. Participants respond when a yellow light and a 2000 Hz tone occur simultaneously. This version includes at least nine practice stimuli and 48 test stimuli, with 16 requiring a response, lasting about nine minutes. It also measures Cognitive Reaction Time (RT/S3 COG) and Motor Reaction Time (RT/S3 MOT).

*Functional performance:* the degree of disability was investigated in terms of the number of Activities of Daily Living (ADL) and Instrumental Activities of Daily Living (IADL) that participants could still manage without assistance [15,16].

*Physical performance:* both the Short Physical Performance Battery (SPPB) and the Tinetti Performance-Oriented Mobility Assessment tool (Tinetti-POMA) or the Tinetti-scale were performed. SPPB comprises standing balance tests (side-by-side stands, semi-tandem test, and tandem test), gait speed measurement, and timed chair stands test, generating scores between 0 and 12, with higher scores indicating better physical performance. The Tinetti Scale assesses motor performance with regard to balance and gait: the scores for each part, a maximum 16 of for balance, and 12 for gait, are combined to obtain an overall score (maximum 28) [17,18].

*Cerebellar volume measurement:* As described in previous work from our group [13], high-definition anatomical images of the cerebellum were collected by means of magnetic resonance imaging (MRI) performed on a 3.0 T scanner (Ingenia, Philips Medical Systems, Best, Netherlands) equipped with a 32-channel head coil. High-resolution T1-weighted (T1w) images were acquired using a 3D-TFE sequence with compressed sensing of 3.5, with TR =  6.7 ms, TE =  3.0 ms, flip angle =  8°, FOV =  240 ×  240 mm^2^, and 1 mm isotropic resolution. N4 bias field correction [19] was applied to the T1w images using the Advanced Normalization Tools (ANTs – [20]), from which cerebellum and brainstem masks were derived. These masks were then used to isolate only the anatomical structures of interest, and any spurious voxels remaining from the masking process were removed using a custom Matlab script (Mathworks, Natick, MA, USA; r2022a update 3 (9.12.0.1975300)). The Spatially Unbiased Infra-Tentorial (SUIT – [21]) Matlab toolbox was used to create a bounding box for cerebellum isolation using the T1w image, by cropping and registering it to the SUIT template in MNI space available with the toolbox through affine and nonlinear transformations using ANTs. The inverse transformation was then applied to the SUIT anatomical parcellation of the cerebellum, bringing it into the subject’s T1w space. Expert neuroradiologists visually inspected the resulting masks to ensure the correct inclusion or exclusion of voxels in the cerebellar parcellations, by overlaying them onto the native structural T1w images, and manually correcting the resulting masks, where necessary. Since the SUIT toolbox includes white matter in its anatomical parcellation, but the regions of interest pertain only to gray matter, individual white matter masks were generated using FreeSurfer 7.1 (https://surfer.nmr.mgh.harvard.edu/) [22]. Brain extraction from the T1w images was performed with ANTs in order to estimate the affine transformation to the brain image obtained from FreeSurfer and was then applied to the cerebellar parcellation. The white matter mask was then used to isolate grey matter tissue in the parcellation. The volume of single cerebellar lobules was computed with an ad-hoc code written in Matlab.

*Ethics approval:* The project received approval from the Ethics Committee for Clinical Experimentation of the Province of Padua (code 5234/AO/21) and was performed in line with the principles of the Declaration of Helsinki. The capacity to provide consent was assessed through a structured process outlined in the written informed consent form, which was specifically designed for this study. Participants were provided with detailed information about the study, its objectives, potential risks, and benefits, and were given the opportunity to ask questions before consenting. In addition, participants were required to demonstrate their understanding of the study’s purpose and procedures before signing the consent form. This was done in the presence of a trained research staff member, ensuring that the participant was able to make an informed and voluntary decision.

### 2.1. Statistical analyses

Cerebellum has been divided into cognitive, motor, and cognitive-motor lobules according to previous findings [11]. Participants’ characteristics were summarized using means ±  standard deviations for normally distributed quantitative variables and medians for variables with non-normal distributions. Normality was assessed using the Shapiro-Wilk test. Categorical variables were presented as frequencies and percentages. To verify the presence of potential differences based on the median of total gray matter cerebellar volume (GMCtv), we stratified participants into two groups using values of GMCtv standardized for participants’ eTIV. Comparative analyses between higher and lower GMCtv groups were conducted using Student’s t-test and Mann-Whitney test for continuous variables, and Chi-square test and Fisher’s exact test for categorical variables. Correlations were assessed using Pearson’s correlation coefficient (r) or Spearman’s rank correlation coefficient (rs) when variables were not normally distributed. To mitigate the risk of Type I error from multiple comparisons, we applied the Bonferroni correction. Multiple linear regression, employing a stepwise forward procedure, was utilized to explore the relationship between physical and performance tests and variables of interest. Statistical significance was considered at p <  0.05. Analyses were conducted using R version 4.1.1 (2021-08-10) (R Foundation for Statistical Computing, Vienna, Austria). The materials and analysis code for this study are not available, and the study was not preregistered.

## 3. Results

The participants’ descriptive characteristics are shown in Table 1. Among the 36 participants, 25 (69.4%) were women, with an average age of 77.78 ± 4.34 years. The mean years of education and comorbidities were 7.56 ± 3.52 and 1.92 ± 1.78, respectively. In terms of neuropsychological assessment, the Mini Mental State Examination (MMSE) score averaged 24.58 ± 4.05. Median scores for ADL and IADL were 100 and 75.00, respectively. Tinetti equilibrium and gait assessments yielded median scores of 16.00 and 12.00, respectively, indicating overall good physical function and mobility within the sample.

**Table 1:** Descriptive characteristics of the sample.

Variables		Total (n = 36)
**Age [years]**		77.78 ± 4.34
**Gender**	Male	11 (30.6%)
	Female	25 (69.4%)
**Scholarity [years]**		7.56 ± 3.52
**Alcoholic units per week**		3.99 ± 4.82
**Retirement age**		60.47 ± 8.86
**Comorbidities [number]**		1.92 ± 1.78
**Marital status**	Married	14 (38.89%)
	Widowed	19 (52.78%)
	Separated/divorced	3 (8.33%)
**Living situation**	With family	20 (55.56%)
	Family + home caregiver	2 (5.56%)
	Alone	14 (38.89%)
**Social isolation**		12 (33.33%)
**MMSE**		24.58 ± 4.05
**Antropometry and Nutritional Status**	BMI [Kg/m^2^]	25.01 ± 3.95
	MNA	25.00 ± 3.01
**Activities of Daily Livings**	ADL	100 (87.49;100.00)
	IADL	75.00 (62.50;100.00)
**Performance Indicators**	SPPB gait speed	4.00 (3.00;4.00)
	SPPB sit-to-stand	2.50 (1.00;4.00)
	SPPB total	10.00 (8.00;12.00)
	Tinetti equilibrium total	16.00 (15.00;16.00)
	Tinetti gait total	12.00 (11.00;12.00)

*Notes*: Values are expressed as mean ±  standard deviation for continuous variables and as frequency (percentage) for dichotomous variables. *Abbreviations*: MMSE =  Mini Mental State Examination; BMI =  Body Mass Index; MNA =  Mini Nutritional Assessment; ADL = Activity of Daily Living; IADL =  Instrumental Activity of Daily Living; SPPB =  Short Physical Performance Battery.

The cerebellar lobules volumes for the entire sample were previously reported in a prior publication and are summarized in S1 Table.

Table 2 illustrates the outcomes of the functional assessment categorized by the median GMCtv. There were no statistically significant differences between those with higher or lower GMCtv in the ADL% [100.00 (83.33;100.00) vs. 100.00 (95.83;100.00)], IADL% [77.50 (61.87;100.00) vs. 75.00 (62.50;100.00)], SPPB total test [10.00 (6.75;11.25) vs. 10.00 (8.00;12.00)], Tinetti equilibrium test and Tinetti gait test.

**Table 2:** Participants’ descriptive functional characteristics stratified according to the median of GMCtv in mm^3^.

Variables	Minor GMCtv (n = 18)	Major GMCtv (n = 18)	p-value	Adjusted p-value
**ADL [%]**	100.00 (83.33;100.00)	100.00 (95.83;100.00)	0.84	0.86
**IADL [%]**	77.50 (61.87;100.00)	75.00 (62.50;100.00)	0.91	0.97
**SPPB – gait speed**	4.00 (3.84;5.00)	4.00 (3.00;5.00)	0.77	0.46
**SPPB - sit to stand**	13.65 (11.25;17.00)	12.00 (10.00;17.25)	0.94	0.95
**SPPB total**	10.00 (6.75;11.25)	10.00 (8.00;12.00)	0.63	0.80
**Tinetti equilibrium total**	16.00 (15.00;16.00)	16.00 (14.75;16.00)	0.96	0.92
**Tinetti gait total**	12.00 (10.25;12.00)	11.50 (10.50;12.00)	0.67	0.86

Notes: Values are expressed as median (interquartile range) or counts (percentages) as appropriate.

*Abbreviations:* ADL = Activity of Daily Living; IADL =  Instrumental Activity of Daily Living; SPPB =  Short Physical Performance Battery.

**Fig 1:**
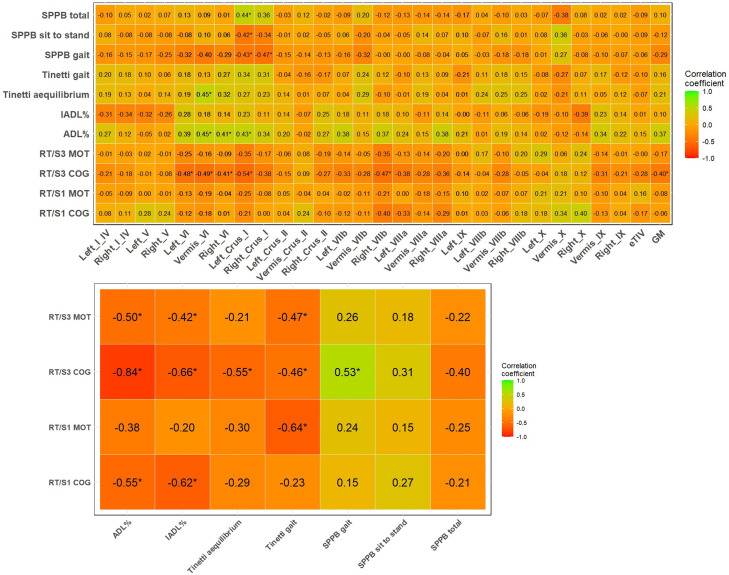
Simple linear correlations between cognitive cerebellar volumes, Reaction Times and performance features. Notes: Correlations were assessed using Pearson’s correlation coefficient (r) for variables that were normally distributed or Spearman’s rank correlation coefficient (rs) when variables were not normally distributed. Abbreviations: ADL =  Activity of Daily Living; eTIV =  Estimated Total Intracranial Volume; GM =  Grey Matter cerebellar volume; IADL =  Instrumental Activity of Daily Living; S1 cog =  Time reaction, first test, cognitive performance; S1 mot =  Time reaction, first test, motor performance; S3 cog =  Time reaction, third test, cognitive performance; S3 mot =  Time reaction, third test, motor performance; SPPB =  Short Physical Performance Battery. Asterisks *  are referred to p < 0.05.

Upon examining the correlations between cerebellar lobule volumes, RTs tests, and performance/functional features, a significant association was found. Specifically, SPPB total (r = 0.44, p = 0.02), SPPB sit-to-stand (r=−0.42, p = 0.01), and SPPB gait speed (r=−0.43, p = 0.01) were significantly correlated with Left_Crus_I. Additionally, SPPB gait speed showed a negative correlation with Right_Crus_I (r=−0.47, p = 0.002), while Tinetti equilibrium was positively correlated with Vermis_VI (r = 0.45, p = 0.003). In terms of ADL scores, significant correlations were observed with Vermis_VI, Right_VI, and Left_Crus_I. Regarding RTs, only RT/S3 COG exhibited a significant negative correlation with entire lobule VI and Left_Crus_I (r=−0.54) (Fig 1). ADL% and IADL% showed negative correlations with RT/S1 COG (r=−0.55 and r=−0.62, respectively), RT/S3 COG (r=−0.84 and r=−0.66, respectively), and RT/S3 MOT (r=−0.80 and r=−0.42, respectively). Additionally, RT/S3 COG exhibited correlations with Tinetti equilibrium, Tinetti gait, and SPPB gait. eTIV was not correlated with RTs and performance variables, while GMCtv was negatively correlated (r=−0.40) with RT/S3 COG.

**Fig 2:**
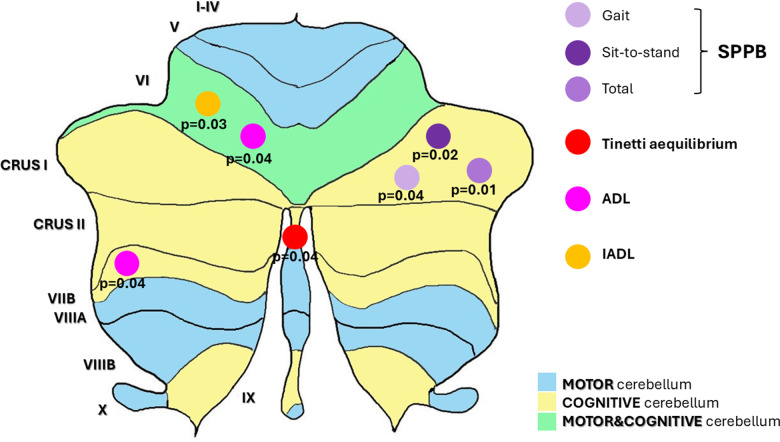
Statistically significant correlations between cerebellar regions and motor areas and cognitive performance: Results of multiple regression analysis. Abbreviations: ADL =  Activity of Daily Living; IADL =  Instrumental Activity of Daily Living; SPPB =  Short Physical Performance Battery.

Table 3, S2 Table and Fig 2 present the results of multiple linear regression analyses examining the associations between physical performance variables and covariates, with particular focus on cerebellar volumes and RT. The regressions were adjusted for gender, age, comorbidity, and MMSE. Across all performance variables analysed, significant associations were observed with RTs. Specifically, RT/S3 COG showed to be relevant in the prediction of SPPB gait speed (R^2^ of the model, p-value of the regression coefficient of the selected variable - R^2^ = 0.44, p = 0.03), as well as in the prediction of SPPB total (R^2^ = 0.46, p = 0.02), Tinetti equilibrium (R^2^ = 0.53, p = 0.04), Tinetti gait speed (R^2^ = 0.62, p < 0.001), and ADL% (R^2^ = 0.78, p < 0.001). The motor component, especially concerning the RT/S1 test, was significant for the prediction of SPPB total, Tinetti gait speed, and IADL%. Furthermore, regarding cerebellar volumes, Right_Crus_I was significant for the prediction of all SPPB tests, while Left_VI for both ADL% (R^2^ = 0.78, p = 0.04) and IADL% (R^2^ = 0.61, p = 0.03). No associations were found between performance variables and eTIV.

**Table 3:** Multiple linear regressions between physical-function variables, cerebellar volumes, and reaction times.

Variables	Predictors	p-value
SPPB gait	RT/S3 COG	**0.03**
	Right_Crus_I	**0.04**
	Vermis_Crus_II	0.07
	Left_VIIb	0.07
	Vermis_IX	0.09
	*R* ^2^	*0.68*
	***ADJUSTED*** *R*^2^	*0.44*
SPPB sit to stand	RT/S1 COG	**0.03**
	Right_Crus_I	**0.02**
	*R* ^2^	*0.34*
	***ADJUSTED*** *R*^2^	*0.25*
SPPB total	RT/S1 MOT	**0.02**
	RT/S3 COG	**0.02**
	Right_Crus_I	**0.01**
	*R* ^2^	*0.65*
	***ADJUSTED*** *R*^2^	*0.46*
Tinetti aequilibrium	RT/S1 MOT	*0.22*
	RT/S3 COG	*0.04*
	Vermis_VI	*0.20*
	Vermis_VIIB	*0.04*
	*R* ^2^	*0.65*
	***ADJUSTED*** *R*^2^	*0.53*
Tinetti gait speed	RT/S1 COG	**<0.001**
	RT/S1 MOT	**<0.001**
	RT/S3 COG	**<0.001**
	RT/S3 MOT	0.06
	*R* ^2^	*0.74*
	***ADJUSTED*** *R*^2^	*0.62*
ADL%	RT/S1 COG	**0.03**
	RT/S3 COG	**<0.001**
	Left_VIIb	**0.04**
	Left_VI	**0.04**
	*R* ^2^	*0.83*
	***ADJUSTED*** *R*^2^	*0.78*
IADL%	RT/S1 COG	**0.01**
	RT/S1 MOT	**0.02**
	RT/S3 MOT	0.06
	Left_VI	**0.03**
	*R* ^2^	*0.73*
	***ADJUSTED*** *R*^2^	*0.61*

Abbreviations: SPPB =  Short Physical Performance Battery; RT/S1 MOT =  Reaction Time, first test, motor performance; RT/S3 MOT =  Reaction Time, third test, motor performance; RT/S1 COG = Reaction Time, first test, cognitive performance; RT/S3 COG = Reaction Time, third test, cognitive performance; ADL =  Activity of Daily Living; IADL =  Instrumental Activity of Daily Living.

Significant p-values are in bold.

## 4. Discussion

In our investigation, we delved into the intricacies of physical and functional performance variables and their correlation with cerebellar lobules volumes and reaction times (both cognitive and motor components). Findings highlight that in walking speed, balance tests, and maintaining daily autonomy, the cerebellar cognitive role is predominant rather than the motor one. In fact, the variability in physical and functional performance tests appeared as significantly associated with the cognitive component of reaction times and the volume of cerebellar lobules involved in cognitive control, corroborating the view of physical performance and cognitive functions as closely entangled.

During the aging process, our health-related behaviors and age-related biological changes often contribute to a natural decline in our physical abilities, which in turn can negatively impact cognitive functions over time [23]. Several studies have highlighted that the prefrontal cortex, parietal lobe, and hippocampus play a key role not only in cognitive function, but also in individuals’ physical performances [24–26]. Current physical activity guidelines suggest that engaging in multi-component physical activity, including balance training, aerobic exercises, and muscle-strengthening activities, could improve cognitive functions and reduce the risk of dementia [23,25,27]. This occurs through improved cerebral circulation, modulation of brain metabolism, promotion of synaptic plasticity and brain function, and reduction of inflammation [28–30]. For example, exercise helps protect neurons in the amygdala and hippocampus from Alzheimer’s-related damage, possibly by enhancing BDNF signaling pathways and promoting the clearance of Aβ [31]. Treadmill training improves memory linked to the hippocampus and supports the dendritic growth of CA1 and CA3 neurons. It also restores memory associated with the amygdala and the dendritic structure of basolateral amygdalar neurons in Alzheimer’s mice [31]. Similarly, physical exercise increases the levels of phosphorylated tropomyosin receptor kinase B (p-TrkB), phosphorylated protein kinase B (p-AKT), and phosphorylated protein kinase C (p-PKC) in both the hippocampus and the amygdala [31]. On the other hand, walking speed relies on the coordination of multiple bodily systems, requiring joints with sufficient mobility, muscles that are strong enough to activate and stop as needed, functional sensory systems for orientation, and an efficient metabolic system to supply energy [3,27]. Consequently, any failure in one or more of these systems tend to manifest in the walking speed of an old individual. In the old population, tests such as the Tinetti scale and SPPB are commonly used to evaluate physical function, including balance, lower limb strength, and walking speed[32]. ADL also describes the difficulty for older adults to perform certain daily self-care tasks, which are essential for maintaining their health and safety [15]. It is known that one of the early signs of dementia is functional deterioration, often manifested by difficulties in performing ADLs. ADLs represent daily activities that challenge both mental and physical capabilities when living independently. A person needs to have the physical ability to independently perform ADL tasks, along with the cognitive capacity to plan and conceptualize these tasks, and these abilities progressively deteriorate with cognitive decline [33]. On the other hand, the SPPB test has been proposed as an appropriate measure for characterizing the frailty status of older adults, as well as a prognostic tool for various adverse outcomes, including cognitive decline [32]. Our findings point toward a correlation between cognitive component of reaction times and motor performance, in both functional and physical tests such as the Tinetti and SPPB (total score) tests. However, what piqued our interest was the engagement of the cerebellar lobule volumes, which not only exhibited significant correlations with physical-functional tests but also manifested associations with them.

The cerebellum is universally recognized as the foremost organ responsible for the coordination of movement: nonetheless, studies on cerebellar lesions are associated with alterations in executive functions, spatial attention, planning, language processes, learning, and memory too [30]. In recent years, there has been increasing attention on the cognitive contribution of cerebellar structure, particularly concerning physical control. Signals from the prefrontal cortex may influence both motor and cognitive outputs in the cerebellum through the fronto-cerebellar network, suggesting its engagement in both domains via this pathway [12,30]. Although evidence regarding the specific roles of individual cerebellar lobules is still limited, a decrease of gray matter in cerebellar lobules V, VI, VIIB, VIIIA, VIIIB, and Crus II together with higher volumes in lobule IV seem to act as predictors of abnormal gait associated with a higher risk of falls in older adults [34]. Our study revealed distinct correlations between cerebellar lobules volumes and gait speed: Right and Left_Crus_I were primarily involved, but only the former highlighted an important association in the regression analysis. In detail, Right_Crus_I maintained an association with all the SPPB parameters. Furthermore, Left_VI, a cognitive-motor lobule, was the only relevant variable in the prediction of ADL and IADL tests, suggesting its potential role in preserving functional autonomy. To date, the role of lobule VI is poorly understood: one study highlighted higher activation in the right portion of lobule VI in healthy individuals standing as compared to Parkinson’s patients, while other pieces of evidence support its involvement in verbal working memory and articulatory movements [35,36].

Motor coordination is crucial in every aspect of the ADL test, as even the simplest activities require precise movement sequences, such as dressing, washing, and eating. In addition to basic activities, the IADL test includes more complex tasks such as managing medical therapies, personal financial management, and meal preparation. Although several studies have investigated the relationship between ADL scores and cognitive abilities, suggesting a negative correlation between cognitive decline and ADL in the older population, specific research exploring the link between cerebellar lobules and the ability to preserve autonomy is currently lacking [37]. It is plausible to hypothesize that deficits in cerebellar coordination activity could compromise the ability to perform such activities, thereby jeopardizing the independence and quality of life of the older adults.

Finally, regarding balance, prior studies have identified regions of the right cerebellar hemisphere and vermian lobules VI-VII, as well as vermian lobules IX-X and hemisphere lobule X of the cerebellum, as potential contributors to balance control [38,39]. However, research on older adults remains limited, leaving an even larger gap in evidence regarding older individuals with mild cognitive impairment. Our study contributes to fill this gap by suggesting balance control as predominantly mediated by cerebellar lobule Vermis VIIb in this population, with results partially reflecting those observed in studies conducted on younger individuals [40,41].

Surprisingly, total cerebral volume (eTIV) was not associated with physical or performance abilities. Previous studies reported that lower brain gray matter (GM) volumes were independently associated with locomotor performance [42–44]. Studies conducted on both healthy individuals and those with cognitive decline have agreed on a positive linear relationship between GM and physical tests [42,43,45,46]. Moreover, a study examined the associations between white matter hyperintensities and walking speed, using eTIV as a reference variable [47]. The results showed that a larger eTIV was correlated with a higher walking speed [47]. These findings were confirmed by other studies, which agreed that smaller brain volumes were associated with slower walking speed [30,48,49]. A positive association between the volumes of the hippocampal and frontal regions and gait speed has been demonstrated [50]. Beauchet and colleagues reported a positive correlation between GM volumes in the left putamen and the right caudate nucleus and gait speed in patients with MCI. However, the authors concluded that the relationship differs between healthy individuals and those with MCI. In healthy individuals, gait speed was positively associated with gray matter volumes in cortical regions, such as the frontal cortex, while in patients with MCI, it was associated with subcortical regions, such as the striatum [51]. Our study reveals the pivotal role of cerebellar cognitive component—rather than motor one—in physical and functional performance, complementing the well-known cerebral control in movement coordination. Exploring previously uninvestigated areas, we disentangled the contribution of the cognitive cerebellum in task traditionally conceived as relying on the motor rather than cognitive cerebellum. Furthermore, the cognitive cerebellar component seems to drive both cognitive and motor component of physical performance test. Altogether, the results bring us back to the provocative question posed by Devita et al., who wondered whether our current understanding of the cerebellum is indeed accurate [11].

### 4.1. Limitations

Several limitations of this study should be acknowledged. Most notably, the absence of a control group confines our findings to individuals with mild cognitive impairment, preventing any conclusions about the potential influence of cerebellar cognitive reserve on clinical trajectories. As a result, this study remains primarily descriptive, highlighting the need for further research to place these findings within the broader context of neurodegenerative disorders, including comparative analyses with healthy cohorts. Additionally, the small sample size necessitates caution in interpreting the results, as they may not fully represent the broader population with mild cognitive impairment. Although in line with preliminary estimation of the sample size, this study is “real-world” based and, therefore, it has both methodological strengths and limitations typical of such approach.

## 5. Conclusions

This study highlighted the importance of cerebellar cognitive functions in the physical and functional performance of older adults with MCI. Significant correlations were found between cognitive reaction times, physical performance, and specific cerebellar lobules such as the Right_Crus_I and left lobule VI, underscoring a close interconnection between these domains. Total intracranial volume showed no association with physical or performance abilities, shifting the attention towards a novel perspective on the cognitive roles of the cerebellum, which may play an unexpected and perhaps more significant role than the brain itself in mediating purely motor tasks. Further research is needed to contextualize these findings within the broader spectrum of neurodegenerative disorders, including comparative analyses with healthy cohorts, to elucidate the specific roles of cerebellar lobules in cognitive and physical functions and their implications for the progression of cognitive impairment.

## Supporting information

S1 Table Participants’ descriptive cerebellar lobules volumes in mm^3^. (DOCX)

S2 Table Multiple linear regressions between physical-function variables and covariates. (DOCX)

S3 Graphical Abstract (JPEG)
