# Supplementary material for: Cognitive cerebellum dominates motor cerebellum in functional decline of older adults with mild cognitive impairment
Source: PLoS One. 2025 Apr 3;20(4):e0321304. doi: 10.1371/journal.pone.0321304 (PMC11967948; doi:10.1371/journal.pone.0321304)
Supplement: S1 Table — (DOCX) [file pone.0321304.s001.docx]

**S1 Table. Participants’ descriptive cerebellar lobules volumes in mm^3^.**

| **MOTOR CEREBELLAR AREAS** | **Total (n=36)** | **COGNITIVE CEREBELLAR AREAS** | **Total (n=36)** |
| --- | --- | --- | --- |
| **Left_I_IV** | *2146.64 ± 431.95* | ***Left_Crus_I*** | 11604.33 ± 1516.17 |
| **Right_I_IV** | *2538.94 ± 563.66* | ***Right_Crus_I*** | 11902.44 ± 1732.54 |
| **Left_V** | *3117.50*  *(2695.25;3312.00)* | ***Left_Crus_II*** | 8255.86 ± 1240.69 |
| **Right_V** | *3369.31 ± 560.87* | ***Vermis_Crus_II*** | 464.56 ± 73.59 |
| **Left_VIIIa** | *4317.33 ± 548.15* | ***Right_Crus_II*** | 7876.08 ± 1139.61 |
| **Vermis_VIIIa** | *949.50*  *(857.25;1053.75)* | ***Left_VIIb*** | 4104.00  (3572.50;4511.50) |
| **Right_VIIIa** | *4069.56 ± 561.96* | ***Vermis_VIIb*** | 181.86 ± 31.85 |
| **Left_VIIIb** | *3602.31 ± 395.79* | ***Right_VIIb*** | 4250.67 ± 625.57 |
| **Vermis_VIIIb** | *512.00*  *(457.75;625.75)* | ***Left_IX*** | 2933.47 ± 411.56 |
| **Right_VIIIb** | *3559.81 ± 454.05* | ***Vermis_IX*** | 662.00  (618.75;721.00) |
| **Left_X** | *694.50*  *(637.00;764.75)* | ***Right_IX*** | 3183.00  (2971.50;3401.50) |
| **Vermis_X** | *364.33 ± 70.41* |  |  |
| **Right_X** | *652.58 ± 111.50* |  |  |
| **COGNITIVE-MOTOR CEREBELLAR AREAS** |  | ***TOTAL VOLUMES*** |  |
| **Left_VI** | *7428.58 ± 1005.42* | ***GMCtv*** | 101464.22 ± 10611.55 |
| **Vermis_VI** | *1724.06 ± 238.43* | ***eTIV*** | 1414266.42 (1353230.99;1528765.00) |
| **Right_VI** | 6892.11 ± 1074.21 | **Vermis-tv** | 4930.81 ± 563.72 |

### *Notes*: Values are expressed as mean ± standard deviation or median (interquartile range) as appropriate.
