# Supplementary material for: Cognitive cerebellum dominates motor cerebellum in functional decline of older adults with mild cognitive impairment
Source: PLoS One. 2025 Apr 3;20(4):e0321304. doi: 10.1371/journal.pone.0321304 (PMC11967948; doi:10.1371/journal.pone.0321304)
Supplement: S2 Table — (DOCX) [file pone.0321304.s002.docx]

**S2 Table. Multiple linear regressions between physical-function variables and covariates.**

| **Variable** | **R^2^** | **Adjusted R^2^** | **p-value** |
| --- | --- | --- | --- |
| **SPPB sit to stand** | **0.34** | **0.25** |  |
| Comorbidities |  |  | 0.0856 |
| GM |  |  | 0.0676 |
| RT/S1 MOT |  |  | 0.0282 |
| Right_Crus_I |  |  | 0.0147 |
| **SPPB gait** | **0.98** | **0.84** |  |
| Sex |  |  | 0.1446 |
| Comorbidities |  |  | 0.0703 |
| MMSE |  |  | 0.0850 |
| eTIV |  |  | 0.0680 |
| GM |  |  | 0.0916 |
| RT/S1 COG |  |  | 0.1582 |
| RT/S1 MOT |  |  | 0.2841 |
| RT/S3 COG |  |  | 0.0373 |
| RT/S3 MOT |  |  | 0.1309 |
| Left_Crus_I |  |  | 0.5964 |
| Right_Crus_I |  |  | 0.0449 |
| Vermis_VIIb |  |  | 0.1551 |
| Vermis_VI |  |  | 0.1922 |
| Vermis_Crus_II |  |  | 0.0688 |
| Left_VIIb |  |  | 0.0748 |
| Right_VIIIb |  |  | 0.1014 |
| Left_X |  |  | 0.1638 |
| Left_V |  |  | 0.1926 |
| Left_VI |  |  | 0.1074 |
| Right_VI |  |  | 0.1128 |
| Vermis_IX |  |  | 0.0914 |
| **SPPB total** | **0.65** | **0.46** |  |
| Sex |  |  | 0.0476 |
| Comorbidities |  |  | 0.0135 |
| GM |  |  | 0.0660 |
| RT/S1 MOT |  |  | 0.0163 |
| RT/S3 COG |  |  | 0.0200 |
| RT/S3 MOT |  |  | 0.2059 |
| Left_Crus_I |  |  | 0.1326 |
| Right_Crus_I |  |  | 0.0131 |
| **Tinetti aequilibrium** | **0.65** | **0.53** |  |
| Sex |  |  | 0.0636 |
| MMSE |  |  | 0.0446 |
| RT/S1 MOT |  |  | 0.2207 |
| RT/S3 COG |  |  | 0.0596 |
| Vermis_VI |  |  | 0.1790 |
| Vermis_VIIb |  |  | 0.0473 |
| **Tinetti gait** | **0.74** | **0.62** |  |
| Age |  |  | 0.05618 |
| Sex |  |  | 0.16743 |
| GM |  |  | 0.04787 |
| RT/S1 COG |  |  | 0.00237 |
| RT/S1 MOT |  |  | 0.00079 |
| RT/S3 COG |  |  | 0.00242 |
| RT/S3 MOT |  |  | 0.05976 |
| **IADL%** | **0.73** | **0.61** |  |
| Age |  |  | 0.02855 |
| eTIV |  |  | 0.04471 |
| GM |  |  | 0.19109 |
| RT/S1 COG |  |  | 0.01110 |
| RT/S1 MOT |  |  | 0.02393 |
| RT/S3 MOT |  |  | 0.06182 |
| Left_VI |  |  | 0.03056 |
| **ADL%** | **0.83** | **0.78** |  |
| MMSE |  |  | 0.1358 |
| RT/S1 COG |  |  | 0.0304 |
| RT/S3 COG |  |  | 4.23e-06 |
| Left_VIIb |  |  | 0.0417 |
| Left_VI |  |  | 0.0388 |

*Abbreviations*: SPPB = Short Physical Performance Battery; GM = Grey Matter cerebellar volume; RT/S1 MOT = Reaction Time, first test, motor performance; MMSE = Mini Mental State Examination; eTIV = Estimated Total Intracranial Volume; RT/S3 MOT = Reaction Time, third test, motor performance; RT/S1 COG= Reaction Time, first test, cognitive performance; RT/S3 COG= Reaction Time, third test, cognitive performance; ADL = Activity of Daily Living; IADL = Instrumental Activity of Daily Living.
