# Supplementary figures and images for: Cognitive cerebellum dominates motor cerebellum in functional decline of older adults with mild cognitive impairment
Source: PLoS One. 2025 Apr 3;20(4):e0321304. doi: 10.1371/journal.pone.0321304 (PMC11967948; doi:10.1371/journal.pone.0321304)

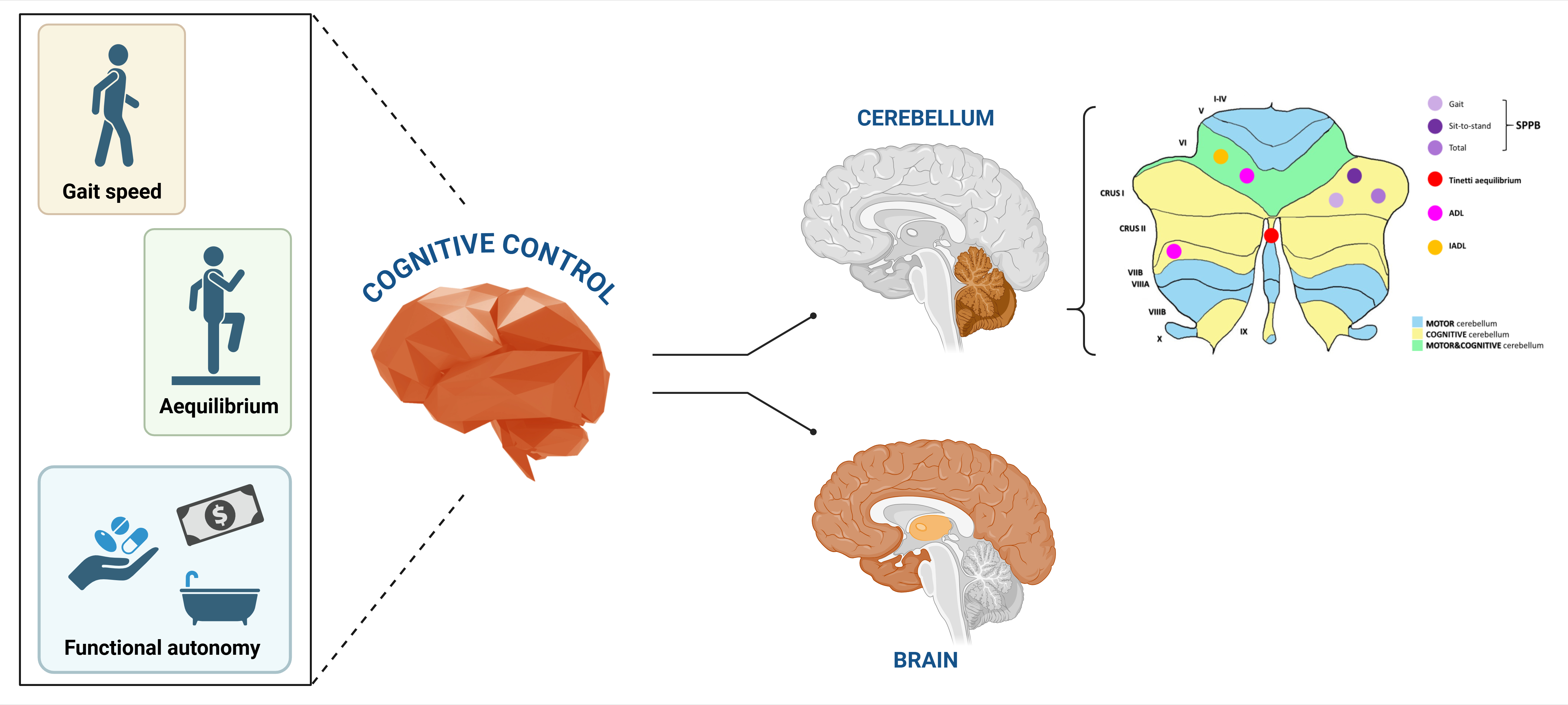

Supplement: S3 Graphical Abstract — (JPEG) [file pone.0321304.s003.jpeg]
